# Multiple pulmonary cavities in an immunocompetent patient: a case report and literature review

**DOI:** 10.3389/fmed.2024.1329381

**Published:** 2024-02-27

**Authors:** Zihan Guo, Anli Zuo, Xinyi Liu, Yunxiu Jiang, Shuran Yang, Degan Lu

**Affiliations:** Department of Respiratory, The First Affiliated Hospital of Shandong First Medical University and Shandong Provincial Qianfoshan Hospital, Shandong Institute of Respiratory Diseases, Shandong Institute of Anesthesia and Respiratory Critical Medicine, Jinan, China

**Keywords:** Legionella, *Legionella pneumophila*, immunocompetent patient, Legionella pneumonia, pulmonary cavity, metagenomic next-generation sequencing

## Abstract

Legionella pneumonia (LP) is a relatively uncommon yet well-known type of atypical community-acquired pneumonia (CAP). It is characterized by a rapid progression to severe pneumonia and can be easily misdiagnosed. In most patients, chest computed tomography (CT) showed patchy infiltration, which may progress to lobar infiltration or even lobar consolidation. While pulmonary cavities are commonly observed in immunocompromised patients with LP, they are considered rare in immunocompetent individuals. Herein, we present a case of LP in an immunocompetent patient with multiple cavities in both lungs. Pathogen detection was performed using metagenomic next-generation sequencing (mNGS). This case highlights the unusual radiographic presentation of LP in an immunocompetent patient and emphasizes the importance of considering LP as a possible diagnosis in patients with pulmonary cavities, regardless of their immune status. Furthermore, the timely utilization of mNGS is crucial for early pathogen identification, as it provides multiple benefits in enhancing the diagnosis and prognosis of LP patients.

## 1 Introduction

Legionella pneumonia (LP) is a severe form of bacterial pneumonia caused by Legionella species. Without appropriate and timely treatment, LP can be life-threatening (1). The mortality rate for LP varies globally, ranging from 5% to 33% among the general population. However, in immunocompromised patients, the mortality rate can exceed 50% (2).

Although the imaging findings of LP are nonspecific, they are closely related to the clinical presentations and outcomes of the disease. Computed tomography (CT) is essential in detecting lung abnormalities, monitoring disease progression, and assessing therapy response. The most common CT pattern observed in LP patients is the presence of well-circumscribed infiltrates with ground-glass opacities, which can involve multiple lobes or segments (3, 4). In immunocompromised patients, it is occasionally observed that abscesses and cavitation may develop during the course of the disease. However, these findings are relatively uncommon in individuals with a healthy immune system (3, 4).

Herein, we present a case of community-acquired LP in an immunocompetent patient who exhibited multiple cavities in the lungs. This case highlights the importance of considering LP as a potential diagnosis in patients with pulmonary cavities, even among those who do not have compromised immune systems.

## 2 Case description

A 67 years-old Chinese female patient was admitted to our hospital presenting with a 5 days history of fever, cough, and exertional dyspnea. She had a productive cough with scant mucus-like sputum. She had no history of smoking and no evidence of *Mycobacterium tuberculosis* infection. On admission, her vital signs were recorded as follows: a body temperature of 39.6°C, a pulse rate of 79 beats per minute, a respiratory rate of 21 breaths per minute, and a blood pressure of 150/84 mmHg. Pulmonary auscultation revealed coarse breath sounds and bilateral rales.

**Figure 1:**
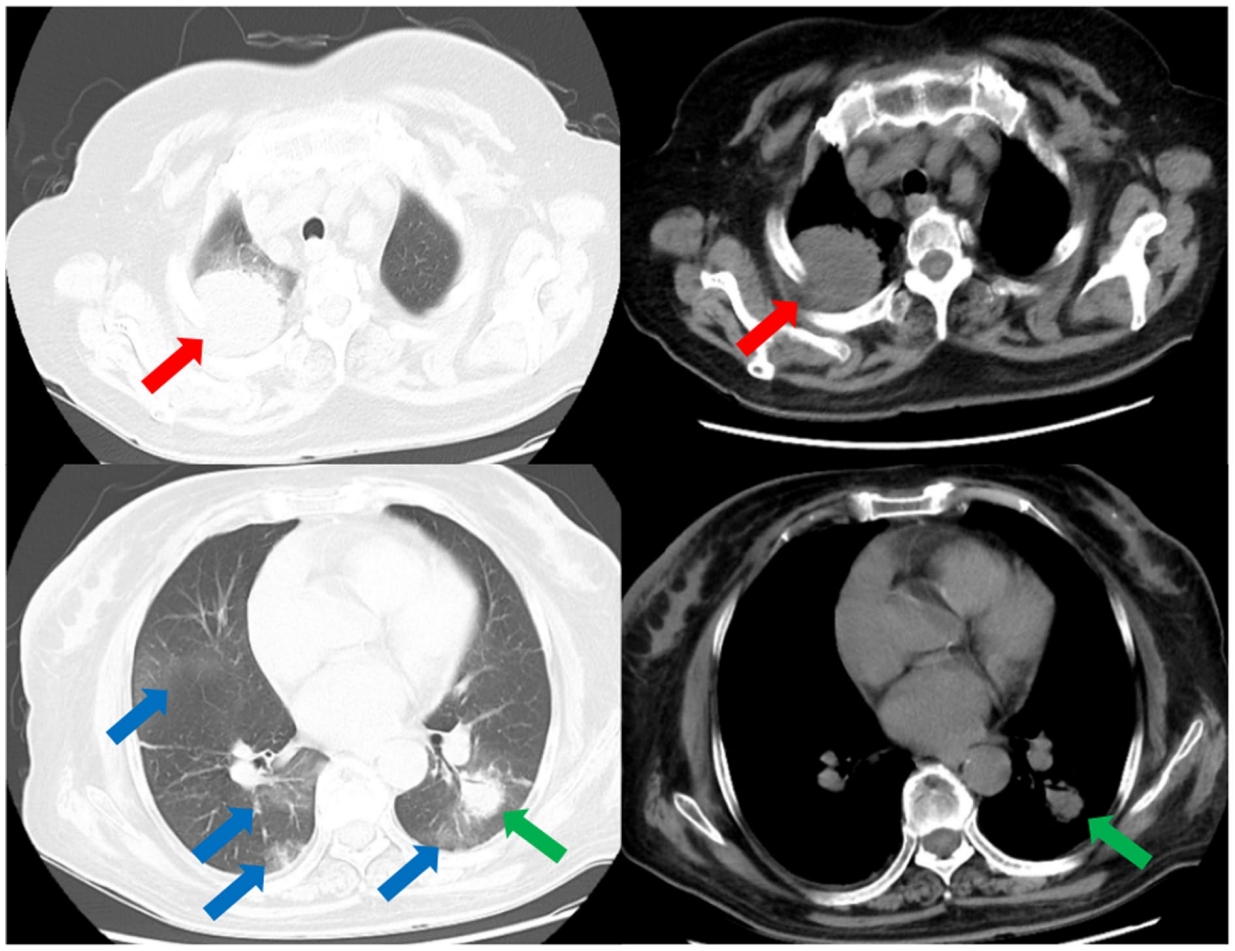
CT scans of the thorax on the day of admission demonstrates multiple nodular and patchy infiltrates in the lungs. Nodular infiltrate in the right lung (red arrows). Nodular infiltrate in the left lung (green arrows). Patchy infiltrates in both lungs (blue arrows).

**Figure 2:**
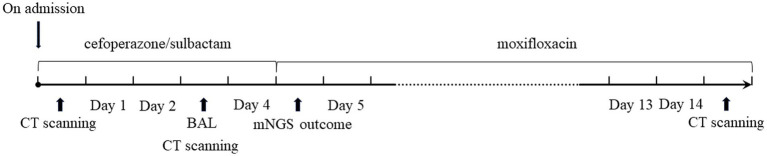
Timeline of treatment process.

Laboratory results upon admission showed a white blood cell count of 14.29 × 10^9^ cells/L (reference range: 3.5–9.5 × 10^9^ cells/L) with an elevated neutrophil ratio of 88%. C-reactive protein and procalcitonin concentrations were markedly elevated at 81.40 mg/L (0–3.48 mg/L) and 1.20 ng/mL (0–0.05 ng/mL), respectively. Serum 1-3-beta-D-glucan and galactomannan tests yielded negative results. CT scans of the patient’s lungs on admission revealed multiple lung nodules and patchy infiltrations (Figure 1). The sequence of relevant events after the patient was admitted has been shown in the timeline (Figure 2).

The patient received empiric treatment with cefoperazone/sulbactam for suspected community-acquired pneumonia (CAP) after blood and sputum samples were obtained. Clinical response assessment after 3 days showed inadequate improvement in the patient’s clinical condition. Microbiological cultures from samples were negative for general bacteria, acid-fast bacilli and fungal elements.

**Figure 3:**
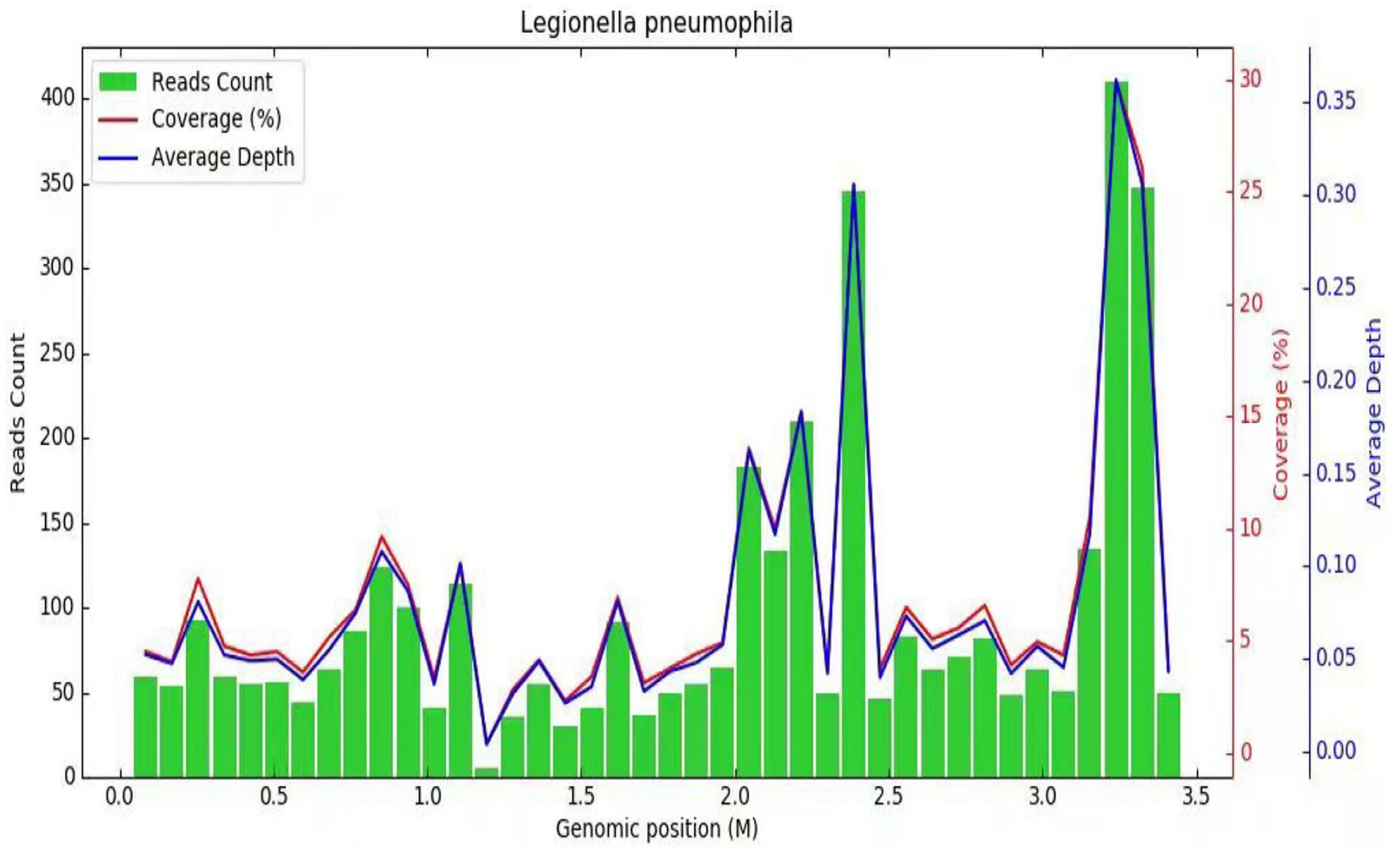
Diagnosis of *Legionella pneumophila* infection using mNGS. The horizontal axis (abscissa) represents the genome of the Legionella bacterium and the vertical axis (ordinate) represents the number of reads detected. The red line indicates the coverage, which represents the ratio of the detected nucleic acid sequence of Legionella to the entire gene sequence of Legionella. The blue line represents the sequencing depth, which is a measure of how many times a specific segment of the Legionella genome has been detected during the sequencing process.

**Figure 4:**
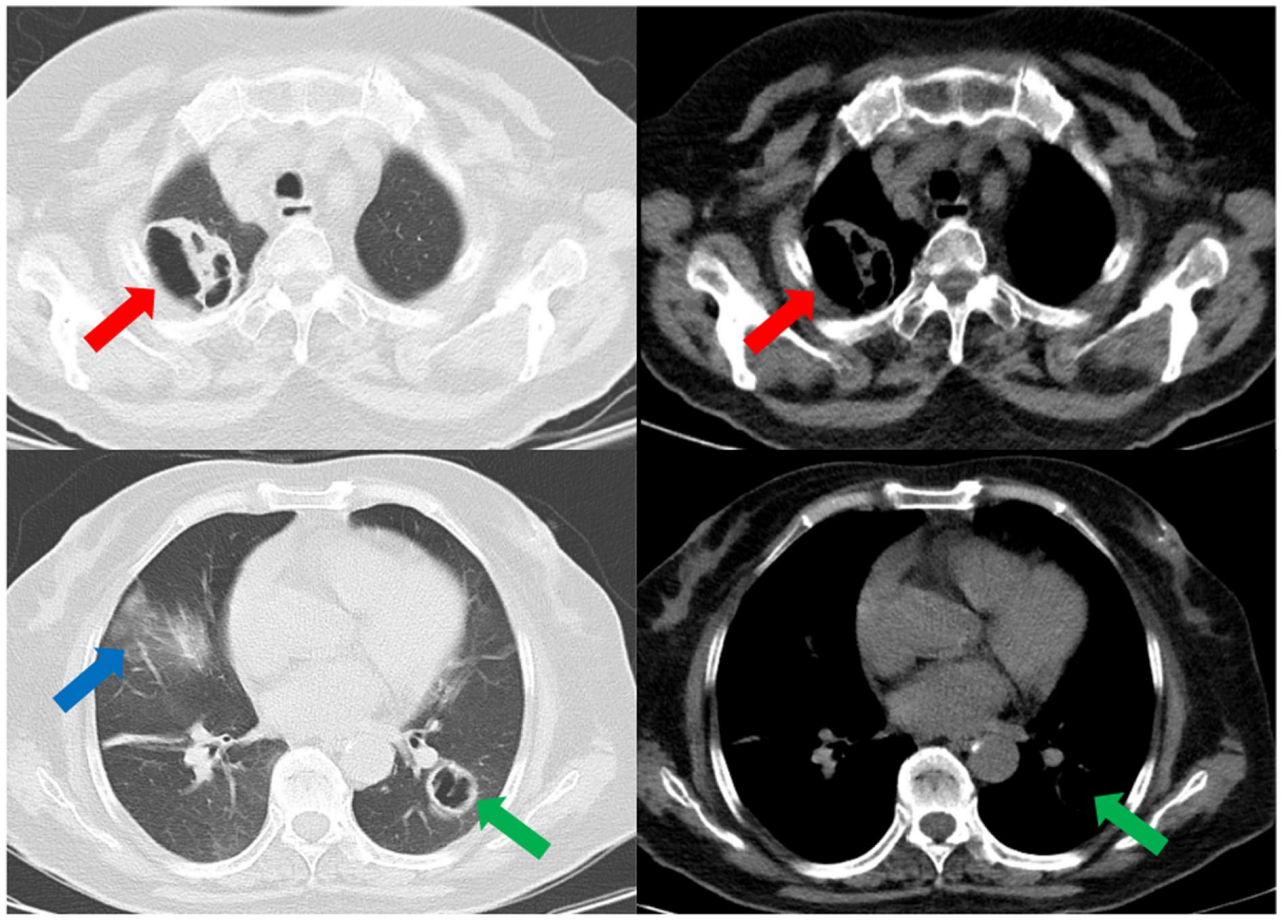
The repeated CT scan conducted on the fourth day after admission showed cavitary lesions in both lungs (red arrows and green arrows). Remaining patchy infiltrate in the right lung (blue arrow).

**Figure 5:**
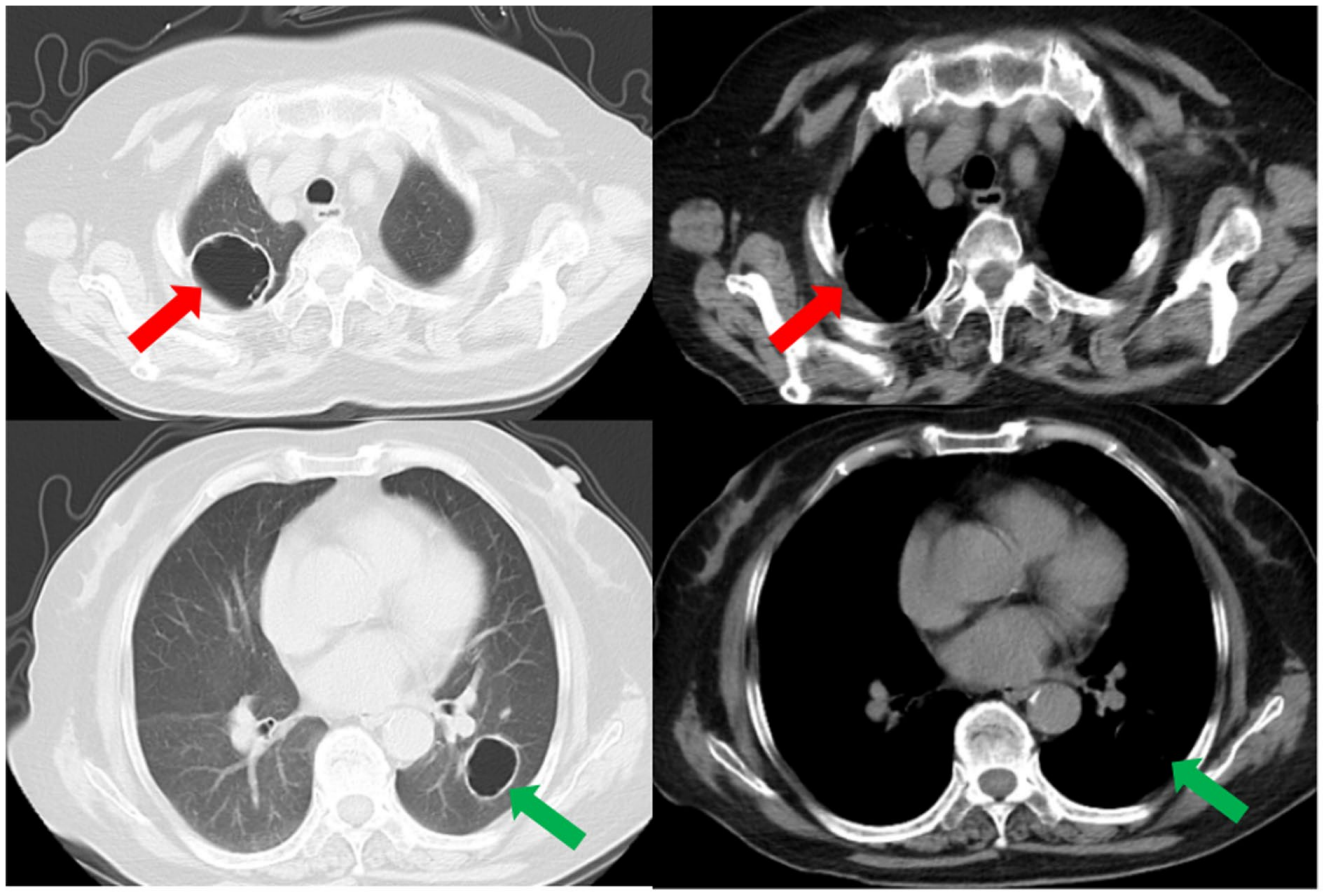
A subsequent CT scan 2 weeks later showed that the cavity in the right lung had decreased in size (red arrow), while the cavity in the left lung had slightly enlarged (green arrow). However, the thickness of the walls of both cavities had diminished, and the bilateral pulmonary infiltration indicated by the blue arrows had been resolved.

Subsequently, a bronchoscopy with bronchoalveolar lavage was performed on the fourth day after admission. Bronchoalveolar lavage fluid (BALF) was sent to Jiangsu Simcere Medical Diagnostics Co., Ltd., and then metagenomic next-generation sequencing (mNGS) was performed on the Illumina next-generation high-throughput sequencing platform. For detailed methodological description, please refer to State Key Laboratory of Translational Medicine and Innovative Drug Development & Jiangsu Simcere Diagnostics Co., Ltd. previously published paper (5). The mNGS results on the 5th day after admission showed that 17,667 original sequences of *Legionella pneumophila* were identified, with a relative abundance of 98.3% and a coverage of 7.22% (Figure 3). Meanwhile, a number of other pathogens have been identified, including certain fungi and bacteria (Table 1). After our analysis, we are more inclined to classify them as respiratory custom flora or background flora derived from the environment or samples, regardless of considering them as pathogenic bacteria. On the same day, a repeated CT scan revealed the presence of bilateral lung cavities (Figure 4). These findings were consistent with LP. Immediate treatment with moxifloxacin injection at a dosage of 0.4 g daily was initiated. After 3 days, the patient’s fever resolved, and there was improvement in cough symptoms. A follow-up CT scan 2 weeks later showed that the cavity in the right lung was smaller. Although the cavity in the left lung was slightly enlarged, the walls of the cavity were thinner, and the infiltration from both lungs had been resolved (Figure 5).

**Table 1:** Reported cases of Legionella pneumonia.

Genus	Number of original sequences	Relative abundance	Species	Number of original sequences
Legionella	17,667	98.30%	*Legionella pneumophila*	17,667
Candida	4	26.67%	*Candida albicans*	4
Pneumocystis	2	13.33%	*Pneumocystis jirovecii*	2
Neisseria	97	0.54%	*Neisseria subflava*	55
Streptococcus	71	0.40%	*Streptococcus australis*	25
Tannerella	29	0.16%	*Tannerella forsythia*	27
Rothia	21	0.12%	*Rothia mucilaginosa*	21
Fusobacterium	19	0.11%	*Fusobacterium nucleatum*	18
Parvimonas	15	0.08%	*Parvimonas micra*	15

Number of original sequences: refers to the number of sequences matching the microbial pathogen at the genus/species level. Relative abundance: the proportion of microorganisms of the same type detected in the entire specimen.

## 3 Discussion and literature review

To begin, a literature search was conducted using relevant databases such as Embase and Medline, spanning up to October 2023. The process carried out during this literature search is further detailed in Supplementary Table S3. After identifying the target papers through this search, the titles and abstracts of the database records were reviewed. Based on this initial screening, the full text of studies that were considered suitable for the evaluation were retrieved. Following the retrieval of the full text, the relevant case data was extracted from these studies.

Among the 14 previously reported cases of LP that were reviewed, 8 patients developed cavitation or abscesses, accounting for 57.1% of the cases (Table 2). Among these 8 patients, 7 were accompanied by immunosuppressive factors, accounting for 87.5%. This suggests that LP patients with immunodeficiency factors are more likely to develop cavities or abscesses, phenomena that are less likely to occur in LP patients with normal immune function. Within the reviewed LP cases, there were 4 deaths (Table 2). In 75% of the fatal cases, the correct pathogen was not identified before the patients’ deaths. Among the 11 surviving cases, 36.4% had Next-Generation Sequencing (NGS) used in diagnosis, and only 27.3% had metagenomic Next-Generation Sequencing (mNGS) used (Table 2). While the application of NGS or mNGS is not yet widespread, all patients who used NGS for LP diagnosis survived and achieved satisfactory results. As evident from the cases reviewed in Table 2, the vast majority of patients received appropriate anti-infective treatment following a correct diagnosis (see Table 3).

**Table 2:** Reported cases of Legionella pneumonia.

Case No.	Gender	Age	Underlying condition/immunosuppression factors	Imaging findings	Diagnosis mode	Therapeutic drugs	Serogroup	Treatment effect
1 (6)	M	52	Good’s syndrome; methylprednisolone 60 mg daily for 2 weeks	Bilateral pleural effusion with patchy shadows and consolidation; pulmonary abscess and cavity formation	Blood mNGS; BALF and soft tissue specimens PCR	FQ, MA	N/D	Good response
2 (7)	F	34	N/D	Interstitial inflammation with multiple lymphadenopathies	tNGS on thoracic lymph nodes specimen	FQ	N/D	Good response
3 (8)	M	31	Smoking history	Consolidation of the right middle and lower lobe	Blood mNGS; culture with BALF	GA, CP, FQ, AG	N/D	Good response
4 (9)	F	34	Liver transplantation	Infiltration progressive cavitation	Urine serotype 1 antigen assay, tissue specimens culture	FQ	I	Good response
5 (10)	F	65	Breast cancer	Patchy consolidation	Blood, sputum, and pleural effusion NGS	FQ	N/D	Good response
6 (11)	F	39	SLE	Cavity, diffuse infiltration & abscess	Serotype 1 antigen assay	MA, LS	I	Good response
7 (12)	M	44	N/D	Alveolar infiltrates, cavitation	Postmortem Pus antigen analysis	BA, MA, AG	I	Bad response
8 (12)	M	31	N/D	Infiltrates	Postmortem tissue antigen analysis	BA, MA, AG, LS	N/D	Bad response
9 (13)	F	39	Breast cancer	Infiltration, cavitation	Culture with BALF, urine serotype 1 antigen assay	MA	I	Good response
10 (14)	M	10	Idiopathic thrombocytopaenia	Round-shaped infiltrates, segmental both-sided pneumonia, a central abcedation inside the infiltrate	BALF PCR& urine by a serotype 1 antigen assay	FQ	I	Good response
11 (15)	F	20	SLE	Patchy infiltrate	Lung tissue autopsy	AG, LS, BA	N/D	Bad response
12 (16)	M	63	N/D	Extensive consolidation	BALF mNGS	FQ	N/D	Good response
13 (17)	F	30	Idiopathic thrombocytopaenia, SLE	Cavitation appeared in consolidation	tracheal aspirates DFA	MA, AG, BA	VI	Bad response
14 (18)	M	45	Chronic myelogenous leukemia	Consolidation, with a abscess formation	Pus biopsy, urine serotype 1 antigen assay	CP, MA, LS	I	Good response

N/D, not determined; MA, macrolides antibiotics; FQ, fluoroquinolone; GA, glycopeptide antibiotic; CP, carbapenem; AG, aminoglycoside; LS, lincosamides; BA, β-lactam antibiotics; F, female; M, male.

**Table 3:** Keywords for searching case reports on abscesses or cavities caused by Legionella and its diagnostic methods.

Medline	(Legionnaires pneumonia) AND (abscess); (legionnaires pneumonia) AND (cavity); (legionnaires pneumonia) AND (Next-Generation Sequencing)
Embase	(“Legionnaires pneumonia” OR (legionnaires AND (“pneumonia”/exp OR pneumonia))) AND (“abscess”/exp OR abscess); (“legionnaires pneumonia” OR (legionnaires AND (“pneumonia”/exp OR pneumonia))) AND cavity; (“legionnaires pneumonia” OR (legionnaires AND (“pneumonia”/exp OR pneumonia))) AND (“high throughput sequencing”/exp OR “high throughput sequencing”)

Based on the reviewed literature and the presented case, there are several noteworthy points that deserve attention and discussion. Firstly, the presence of cavities in the lungs, which is more commonly seen in immunosuppressed patients with LP, was found in an immunocompetent individual. Generally, immunocompetent individuals are more efficient in clearing Legionella bacteria, leading to fewer abscesses and cavities compared to immunocompromised patients (19, 20). In cases where immune function is compromised due to factors such as immune deficiency, organ transplantation, chemotherapy, or prolonged use of immunosuppressants, the immune response may become inadequate, potentially allowing bacterial growth and resulting in abscesses and cavities in the lungs (6, 9, 10, 15, 17, 18). In our patient, investigations for underlying causes of immunosuppression did not reveal any significant findings. When the immune system recognizes the presence of Legionella bacteria, it mounts a strong inflammatory response to eliminate the infection (20). However, this intense immune response can also cause collateral damage to the surrounding lung tissue, leading to the formation of abscesses or cavities (21).

Secondly, mNGS plays an important role in detecting *Legionella pneumophila* based on the analysis of BALF. Since LP lacks specific radiographic features, pathogen evidence seems to be more convincing in diagnosis compared with radiographic findings. In our case, the routine microbiological tests yield negative results and it is difficult to diagnose the causative agent *Legionella pneumophila* using traditional methods. Legionella culture and urine antigen testing are commonly used clinical methods for Legionella detection. However, they have limitations such as time-consuming culture results, sensitivity issues, and potential false positives (3, 22–24). In contrast, mNGS has proven to be effective in identifying challenging-to-culture pathogens like Legionella (25). It utilizes nucleic acid detection and molecular techniques, which offer enhanced sensitivity, specificity, and rapid pathogen detection throughput (26). This technology can be particularly useful in clinical settings for precise diagnosis and timely treatment (27).

Finally, this patient responded well to treatment for LP, indicating the effectiveness of the selected antibiotics in combating the infection. According to the etiological characteristics of Legionella, most macrolides, tetracyclines, ketolides, and quinolones are effective, with good efficacy and relatively few side effects, while Beta-lactams and aminoglycosides are ineffective (3). For severe or life-threatening LP, the British Thoracic Society recommends the use of fluoroquinolones (28). Studies have shown that moxifloxacin, in comparison to levofloxacin, exhibits at least an equivalent anti-Legionella effect and superior pharmacological parameters *in vitro* (29–31), as well as in clinical treatment (32). Notably, moxifloxacin does not require dosage adjustment in patients with liver and kidney dysfunction, making it a preferred choice due to its convenient dosing frequency and feasible administration method. The optimal duration of LP treatment varies based on clinical presentation and individual factors, however, a 2 weeks course of highly active anti-Legionella antibiotics is typically deemed sufficient (24). In severe cases of LP, particularly in patients who are immunocompromised or have failed initial treatment, the treatment duration should be extended to 4–6 weeks (24, 33). While there is still uncertainty about the ideal treatment for cavitary LP, studies have suggested that both macrolides and quinolones have shown efficacy against cavitary disease (34). In general, anti-infective treatment for cavitary LP is recommended for at least 4 weeks or until the lung cavity disappears (35).

## 4 Conclusion

In summary, this case illustrates an unusual form of multiple cavities in both lungs in an immunocompetent patient with LP. mNGS is an advanced diagnostic tool that can be valuable in confirming the diagnosis of Legionella infection and guiding treatment decisions. Either a fluoroquinolone or a macrolide is recommended as a first-line antibiotic treatment for LP, including cavitary LP. Although rare, LP should be considered in the differential diagnosis of cavitary lung diseases, even in immunocompetent patients.

## Data availability statement

The original contributions presented in the study are included in the article/Supplementary material, further inquiries can be directed to the corresponding author.

## Ethics statement

The studies involving humans were approved by Ethics Committee of the First Affiliated Hospital of Shandong First Medical University. The studies were conducted in accordance with the local legislation and institutional requirements. The participants provided their written informed consent to participate in this study. Written informed consent was obtained from the individual(s) for the publication of any potentially identifiable images or data included in this article.

## Author contributions

ZG: Conceptualization, Data curation, Investigation, Writing – original draft, Writing – review & editing, Software, Visualization. AZ: Conceptualization, Investigation, Writing – original draft. XL: Investigation, Writing – original draft. YJ: Software, Writing – original draft. SY: Investigation, Writing – original draft. DL: Funding acquisition, Project administration, Resources, Supervision, Writing – review & editing.
